# Work Disability in Axial Spondyloarthritis

**DOI:** 10.1007/s11926-020-00932-5

**Published:** 2020-07-27

**Authors:** Elena Nikiphorou, Sofia Ramiro

**Affiliations:** 1 Leiden University Medical Center, Leiden, The Netherlands; 2 King’s College London, London, UK; 3 Zuyderland Medical Center, Heerlen, The Netherlands

**Keywords:** Axial spondyloarthritis, Ankylosing spondylitis, Absenteeism, Presenteeism, Work productivity, Sick leave

## Abstract

**Purpose of Review:**

Axial spondyloarthritis (axSpA) is a chronic inflammatory disease that typically affects people of working age. Work-related outcomes are therefore important to study, both from an individual but also a societal perspective. Through this review of the literature, we explore the impact of axSpA on key work outcomes including work and productivity loss and predictors for these.

**Recent Findings:**

Recent evidence confirms that axSpA is associated with substantial consequences on the ability to work. Reassuringly, early treatment and use of biologics have been associated with improved wok outcomes highlighting the importance of prompt diagnosis and management. High disease activity, labour-intensive jobs, poor physical function and impaired spinal mobility are among identified predictors of adverse work outcomes in axSpA.

**Summary:**

The impact of axSpA on work outcomes is considerable and necessitates optimal intervention, including suppression of disease activity, to enhance people’s chances of remaining in work.

## Introduction

Spondyloarthritis (SpA) is a chronic inflammatory disease that can affect the axial skeleton (axSpA) and/or the peripheral joints. AxSpA can have radiographic evidence of sacroiliitis fulfilling the New York criteria (radiographic axSpA, r-axSpA [1], what is classically referred to as ankylosing spondylitis [2], or it can be without radiographic sacroiliitis (non-radiographic axSpA, nr-axSpA) [3]. Large variations are seen in reports on prevalence rates of SpA, ranging from 0.20% (95% confidence interval [95% CI] 0.00–0.66) in South-East Asia to 1.61% (95% CI 1.27–2.00) in Northern Arctic communities [4]. These variations are thought to be driven by differences for example in the mean age of samples studied, source population and geographical areas (also reflecting a different prevalence of HLA-B27 positivity), differences in social security systems and different labour markets, year of data collection and the study methodology [4, 5].

Aside from spinal inflammation, peripheral arthritis and dactylitis, extra-articular manifestations such as uveitis, psoriasis and inflammatory bowel disease, can add to the burden of axSpA. A number of other conditions can also co-exist with axSpA, classically referred to as comorbidities. These include metabolic bone and cardiovascular disease [6–8] which further contribute to the complexity of patients, challenging their management and outcomes. AxSpA has been associated with severe physical limitation, functional impairment and decreased quality of life [9, 10]. These, along with work-related outcomes, are key patient-reported outcomes (PROs) that more pragmatically reflect the impact of the disease on a patient’s life and enable a more holistic patient management that takes into account aspects that matter most to patients.

Being a disease of young people typically of working age inherently makes work ability and productivity an important outcome of interest. Evidence suggests that the impact on work productivity can be enormous [11]. In fact, several studies over the years which have addressed this important issue have demonstrated substantial consequences on the affected persons’ ability to work. In this review, we summarise the impact of axSpA on work ability, particularly focussing on more recent studies, in an attempt to understand the scale of the problem and potential factors affecting work-related outcomes such as disability and productivity loss.

## Employment Rates and Work Productivity in axSpA

### Loss of Employment

Compared to the general population, employment rates in r-axSpA are decreased, especially in male patients, whereas work disability rates are substantially increased. In a study of patients from the nationwide Dutch Standard Diagnosis Register of Rheumatic Diseases (SDR) [37 rheumatology practices (66%)], withdrawal from work was three times higher in patients with r-axSpA compared to the general population [12]**.**

Variations in the reported rates of work disability at least for studies completed in earlier years are inevitable and as highlighted above, not only due to differences in underlying work structures, social insurance and policy systems, but also due to methodological differences across studies, definitions (e.g. for work-related outcomes) used and types of populations examined [13]. The reported rates of work disability in SpA have been historically high. For example, based on data from France, cumulative withdrawal rates of 36% are reported, in those with 20-year disease duration [14]. In Finland, withdrawal rates of 5% after 10 years of disease are reported, 30% after 25 years [15]. More recent data continue to demonstrate the scale of the problem. For example, French cross-sectional data from a tertiary care centre using a work instability self-administered scale (AS-WIS) that can identify patients experiencing a mismatch at work who are risk of job loss [16] suggested rates of 35% and 5% for moderate and high work instability, respectively [17]. Based on Spanish cross-sectional data, the reported permanent work disability in r-axSpA is 26% [18]. Turkish data based on at least 12-month follow-up in patients with r-axSpA report that 45% of patients had changed to a lighter job and 24% became retired due to their disease [19]. The mean retirement age in the Turkish study was 36 ± 4.2 years, with frequency of hip involvement being higher in the work-disabled group, in support of previous data [12]. Cross-sectional data from across 17 regions in Italy show that 21% of patients had to change or leave their job or had actually lost it due to their SpA [20]. The employment rate was 53% (compared to 58% in the general population), after correction for the higher percentage of male patients in the study compared to the general population.

Outside Europe, data from the US based on the observational Prospective Study of Outcomes in Ankylosing Spondylitis (PSOAS) with patients recruited between 2002 and 2007 reported 13% of patients with r-axSpA being work disabled compared to the expected work disability rate of 6% in the general population [13]. Ninety percent of patients who were work disabled reported that this was due to their disease with potential predictors for work disability including socio-economic factors (see below).

However, recent times are showing promise in the outlook of axSpA also in terms of work-related outcomes. Specifically, data from the British Society for Rheumatology Biologics Register (BSRBR-AS) from across at least 80 centres and with a large study population (*n* = 1188 [62%] in paid employment at recruitment) demonstrate that leaving employment, the worst work outcome, is in fact relatively low, with only 52 people (4.4%) reporting leaving work during follow-up while still in working age [21••]. In another study from the BSRBR-AS, including a meta-analysis of 1109 subjects across observational studies and trials, treatment with biologics was associated with significantly greater improvements in work productivity and activity impairment (see below). These findings demonstrate the potential of biologic therapies in axSpA to reduce adverse work-related outcomes and highlight the need for prompt diagnosis and treatment.

### Absenteeism

Substantial absenteeism or sick leave has also been described in patients with axSpA [11] with correlations between decreased work productivity and increased disease-related sick leave [11, 22]. For example, in a Dutch longitudinal study of patients with r-axSpA from the Outcome in AS International Study (OASIS) cohort (mean [SD] disease duration 14.9 [9.3] years), 12% of those in paid employment (51%) were reported to have had sick leave over a period of 2 weeks and 53% experienced an adverse effect of the disease on work productivity while at work [11]. Another Dutch study in patients with early SpA (< 5-year disease duration) showed sick leave rates of 28% in a specific area in Amsterdam [23], almost 7 times higher than in the general population in Amsterdam and also higher compared to other data.

Based on data from the Italian section (population) of the Spondyloarthritis Caught Early (SPACE) cohort, absenteeism was very low compared to that in patients in other countries [24•]. The study identified 7.9% of absenteeism in patients with paid work, a finding that was similar to the sick leave in the general Italian population (8.4%). In another Italian, cross-sectional study based on the ‘ATLANTIS’ survey, more hours were reported as having been lost over the previous 7 days due to SpA (on average, 2.39 h vs 1.67 h), than for other reasons, e.g. holidays/family commitments. The hours of absence due to the SpA was 59% of the hours of absence from the work place and 7% of weekly working hours. Over a third of patients in this study reported important limitations that impacted on their professional development and career [20].

The differences in reported rates of absenteeism and other work-related outcomes seen across studies, as also discussed higher up, could be due to multiple reasons including methodological variations across studies, differences in sick leave regulations and data recording, year of the study, different patient demographics, patient characteristics (e.g. presence of radiographic vs non-radiographic disease, disease duration). Data on sick leave thus vary widely in the literature, with clear differences across countries, e.g. 16% in the UK, 48% in France and 52% in the Netherlands [25–27].

Reduced work ability leading to sick leave is therefore a common consequence of axSpA [28] that should not be ignored especially as it can also have socioeconomic impact. For example, among patients who report sick leave, health-related resource utilisation is reported to be higher [23]. The psychosocial and financial impact on both the individual affected, as well as the society as a whole, can thus be considerable [12, 25].

### Presenteeism

Although one of the most extreme impacts of axSpA on work outcomes is work cessation or (less so) the need to change jobs due to limitations of the disease, axSpA has also been associated with inability to perform one’s job adequately, i.e. presenteeism. Evidence suggests that the levels of presenteeism can be higher than absenteeism (22–33% vs 5–9%), a finding that is seen across different countries [29, 30]. Recent, multi-centre data from the BSRBR-AS based on 1188 participants who were working at recruitment confirm these observations with 79% of participants having reported some presenteeism compared to 19% absenteeism in the past week, as a result of their axSpA [21••].

The chronic nature of the disease and more importantly, the onset of symptoms at an early age, affecting social and work participation can have devastating consequences both on the individuals affected, their family, carers and society as a whole. Studies report significant restrictions in paid and unpaid work. Among patients with r-axSpA in an unselected prevalence cohort with longitudinal follow-up (OASIS cohort), 71% had experienced restrictions in unpaid work during the previous 2 weeks [11]. Significant annual production costs were also reported, up to 1930 Euro (95% CI 1404–2471) to substitute loss of unpaid work production [11]. Although the specific study was based on a small sample of data from a single centre in The Netherlands, these findings, along with those from studies in other countries at larger scale [21••], support the importance and relevance of considering presenteeism when assessing work impact in axSpA.

## Factors Associated with Adverse Work Outcomes in axSpA

Different factors have been associated with adverse work-related outcomes in axSpA. Work productivity for example is reported to be reduced more in women and to be associated with worse quality of life, disease activity, physical function, self-efficacy and depression in a cross-sectional study in south Sweden [26]. These findings are in line with other data reporting psychological (e.g. depression), sociodemographic (e.g. social deprivation) and disease-related factors to be associated with work status [27]. Depression in particular has been strongly associated (OR 5.7, 95% CI 1.8–18.3) with employment status, absenteeism and presenteeism, emphasising the need for addressing this comorbidity in routine clinical practice. Older age, lower social class (lower educational level and manual, physically demanding jobs) and unfavourable coping strategies all identified as important determinants of work withdrawal [12]. Comorbidities and having had a total hip replacement (THR) were also associated with being work disabled [12].

Perhaps not surprisingly, work-related outcomes can be inter-connected. For example, work cessation (leaving employment) based on multi-centre data from the BSRBR-AS [21••] was most strongly associated with a history of previous absenteeism (RR 1.02 per % increase in absenteeism, 95% CI 1.01–1.03), which itself was associated with prior presenteeism and having a labour-intensive job (presenteeism: 0.14% average increase in absenteeism at follow-up for every % increase in presenteeism at baseline [95% CI 0.07–0.2]; labour-intensive job [2.7, 95% CI 0.4–4.9] [21••]. Other factors associated with presenteeism and absenteeism in data from the BSRBR-AS are shown in Box 1. It is worth noting that work outcomes in this study and as for several studies investigating work outcomes in axSpA were assessed using the Work Productivity and Activity Impairment Specific Health Problem v2.0 (WPAI:SHP) scale [31] validated for use in patients with r-axSpA [32].

**Table:** Box 1 Factors associated with absenteeism and presenteeism based on large registry data from the British Society for Rheumatology Biologics Register in Axial Spondyloarthritis (BSRBR-AS) [21••]

**Absenteeism**	
Work-related factors	
Previous history of presenteeism, labor-intensive job.	
Patient-related factors	
Higher disease activity, poorer physical function, poorer quality of life, activity impairment, spinal pain, fatigue, sleep disturbance	
**Presenteeism**	
Work-related factors	
Labour-intensive job, previous history of absenteeism	
Patient-related factors	
Higher disease activity, poorer physical function, impaired spinal mobility, worse global disease status, poorer quality of life, worse spinal pain, fatigue, sleep disturbance, commencing biologic therapy, peripheral joint involvement	

Several studies (most at cross-sectional level) have shown associations between work productivity impairment and high disease activity and poor functional ability in axSpA [24•]. Data from the UK [29] show that higher fatigue levels were strongly associated with all work measures (absenteeism, presenteeism and work productivity loss) as well as with daily activity impairment, highlighting the relevance of understanding and managing where possible this debilitating symptom. Furthermore, Bath Ankylosing Spondylitis Disease Activity Index (BASDAI) and Bath Ankylosing Spondylitis Functional Index (BASFI) were associated with higher presenteeism and work productivity loss [29]. Data from two tertiary centres in Singapore confirm that active disease, reduced physical function and poorer quality of life are associated with reduced work productivity in axSpA [33•]. More specifically, among employed patients, mean (SD) absenteeism, presenteeism and work productivity loss were 4.5% (13.7), 24.9% (19.9) and 27.6% (23.2), respectively. Absenteeism was significantly associated with disease duration and EQ-5D, whereas presenteeism was associated with BASDAI, BASFI and EQ5D; work productivity loss was significantly associated with BASFI, EQ-5D (*p* < 0.05 for all).

Evidence from large cohort data demonstrate that people with axSpA who are not in employment have worse disease activity and function, as well as poorer quality of life [21••]. Patient/disease-specific characteristics and work-related factors, in particular labour-intensive jobs, disease activity, fatigue and poor function, have all been strongly related to future episodes of presenteeism [21••]. Data from the SPACE cohort, including patients with early axSpA, demonstrate the striking impact of disease activity on work productivity over 1 year in axSpA, with every unit decrease in Ankylosing Spondylitis Disease Activity Score (ASDAS) demonstrated to be associated with improvements in absenteeism (defined as absence at work due to disease), presenteeism (decreased functionality at work due to disease), work productivity loss (total work impairment due to disease [takes into account both presenteeism and absenteeism]) and activity impairment (impairment in daily activities) by 5, 17, 16 and 18%, respectively [30•]. In this study, shorter symptom duration was associated with a higher impact of disease activity on work productivity and the impact of absenteeism was higher in patients initiating drug therapy [30•]. Data from the DEvenir des Spondyloarthrites Indifféren ciées Récentes(DESIR) cohort suggest that sedentary jobs (white-collar jobs) are associated with persistent inactive disease and that persistent high disease activity trajectories were significantly associated with consequences on work, specifically greater declaration of sick leave and work disability over follow-up [34].

Job type per se is not necessarily a reflection of adverse work outcomes (unless this is clearly the case with a change in job situation because of the disease itself). Interestingly, studies have looked into the consequences of job type across cohorts, with consistent findings. For example, the relationship between disease activity and radiographic progression was longitudinally assessed in OASIS and shown to be significantly and independently modified by job type [35]. In line with this, recent data from the DESIR cohort support a strong association between smoking and sacroiliac joint inflammation on magnetic resonance imaging over time in patients with axSpA with a blue-collar job or with low education, irrespective of other socioeconomic factors, systemic inflammation or treatment [36]. Although these studies throw more light into these possible associations, whether these are indeed related to the actual job itself, the socio-economic status or the mechanical stress the job imposes, remains to be unveiled.

In the observational PSOAS study based in the US [13], disease duration had an impact, with the association of r-axSpA with both work disability and non-participation in the labour force being stronger among patients with ≥ 20-year disease duration compared with those < 20 years and stronger among patients aged ≥ 45 years compared with those aged < 45 years. Furthermore, the likelihood of work disability and nonparticipation in the labour force was higher among those with lower education, in line with other evidence [12].

## Impact of Treatment on Work Outcomes in axSpA

### Clinical Trial Data

Clinical trial data can provide useful information when it comes to understanding the impact of drug therapy on work-related outcomes. Double-blind periods of clinical trials can be informative, but for most of work-related outcomes, a longer follow-up is needed to be able to make meaningful conclusions. Therefore, despite the lack of comparator, long-term extensions are here more relevant. In line with this, data from a 2-year open extension study of a randomised placebo controlled trial, in which all patients with r-axSpA were treated with 5 mg/kg infliximab, demonstrate a significant reduction in sick leave from 57 to 36% of all employed patients after 1 year of treatment [37]. In the second year of treatment, the percentage of patients on sick leave was further reduced to 14%. This was significantly different compared with baseline as well as with the first year. There were significant reductions in the mean days of sick leave too, for each patient, further translating to reductions in the direct and indirect costs in patients with r-axSpA [37].

Furthermore, based on data up to 96 weeks from the RAPID-axSpA trial, a phase III, double-blind, multicentre study in patients with axSpA, compared with placebo, patients treated with certolizumab pegol improved productivity at the workplace as well as participation in household and social activities [38•]. What was even more striking in this study is that improvements seen with certolizumab pegol were maintained to week 96, with further reductions seen in the absenteeism and presenteeism due to axSpA and lower interference of axSpA with work productivity. The findings were similar between r-axSpA and nr-axSpA subpopulations [38•].

Several other trials support the positive influence of TNFi on work-related outcomes in axSpA. For example, data from a phase III trial of adalimumab vs placebo in nr-axSpA, ABILITY-1, demonstrated favourable and significant improvements in work productivity [39]. Similarly, data from the phase III GO-RAISE trial assessing golimumab vs placebo demonstrate significant improvements in work productivity as well as HRQOL through 2 years [40].

Generally, the evidence from RCTs on work-related outcomes is often based on subjective patient-driven assessments and it is not the primary objective of the studies, with data reported from the extensions of controlled trials. Still, in the aforementioned trials and others like the ASSERT trial [41] which included a visual analogue scale (range 0–10) to measure the impact of disease on work productivity and data on self-reported employment status and time lost at work before and during the trial, the findings are overall promising although they appear more striking for work productivity-related outcomes. In the ASSERT trial [41], the use of infliximab (compared to placebo) significantly improved productivity and reduced workday loss among employed patients with r-axSpA. In terms of time lost from work, those on infliximab lost fewer workdays during the 6-week period prior to week 24 (0.7 ± 3.5 days) compared with the 6-week period prior to baseline (1.7 ± 4.8 days, *P* < 0.01) unlike the case with the placebo group where no significant reduction was observed. In terms of workdays missed per patient during the 24-week study period, this did not reach statistical significance with the mean number (mean ± SD) being 6.8 ± 17.6 in the placebo group compared with 2.8 ± 13.3 in the infliximab group (*P* = 0.07) [41].

Similarly, in a multicentre, double-blind, randomised phase IIIB clinical controlled trial of nr-axSpA across 14 countries [42], patients receiving etanercept experienced a significantly greater improvement in presenteeism and activity impairment compared to placebo, but not in absenteeism. In the same study, in placebo patients switching to open-label etanercept, responses in work-related outcomes were similar to those who originally received etanercept, with the exception of absenteeism.

### Real-Life Observational Data

Real-life observational data have an important role in demonstrating the impact of disease and treatment on work-related outcomes, also in the longer-term, even though confounding by indication is a recognised problem. Yet, if the design and methodology are adequate, the findings can be particularly informative. Recent data from the BSRBR-AS provide evidence that treatment with bDMARDs significantly improves work productivity and activity impairment in people with axSpA, further supported by pooled estimates from a meta-analysis of BSRBR-AS data combined with other studies [43••]. An earlier, questionnaire-based study from the UK, despite a small sample size, supports these findings with improvements on work capacity (indicated by the ability to return back to work and ability to increase work for example from part-time to full-time and to also work more productively) in patients with r-axSpA following use of TNFi including infliximab, etanercept and adalimumab [44].

Of interest and in line with most of the clinical trial data, the BSRBR-AS study and meta-analysis study [43••] did not show significant improvements with the use of biologics in absenteeism, despite improvements in presenteeism, work impairment and activity impairment. Older studies based on cohort data have reported on absence from paid work, demonstrating reductions with the use of biologics, although this was not always statistically tested.

## Implications and Opportunities

The ability to remain in work has relevance at both a personal and societal impact. Patients with r-axSpA rank the ‘impact on work’ as one of the most affected aspects of their life as a result of their condition [45]. Aside from the impact on the individual’s social and psychological well-being, there are socioeconomical consequences that cannot be ignored [46]. Decreasing disease activity, improving function and spinal mobility among other, can have positive clinical impact in patients with axSpA maximising their chances of staying in work. People with axSpA should have access to appropriate support in the work place to reduce work impairment and prevent damage to their careers and life overall. To date, most of the evidence comes from long-term cohort data often in patients with established r-axSpA, with general scarcity on work-related outcomes in the early phases of disease. Going forward, we hope to see wider recognition of the importance of work-related outcomes in axSpA in both clinical and academic settings, the latter resulting in more research opportunities, also in nr-axSpA.

## Conclusions

AxSpA affects people early, usually in their second and third decade of life, which tend to be the most productive years [5]. Addressing the ability to work is thus crucial in supporting people living with axSpA to remain in the workplace, keeping them also ‘connected’ and engaged socially. Insights into potential predictors of adverse work outcomes can help clinicians better manage their patients with axSpA, reducing their risk of work disability. The evidence over the years has been strong, providing greater insights not just into predictors of poor work outcomes, but also on the importance of early diagnosis and treatment. Yet, considerable unmet needs remain, calling for more active research as well as greater public awareness in this topic. Initiatives like the EULAR Task Force on ‘Points to Consider when designing, analyzing and reporting studies with work participation as outcome among patients with inflammatory arthritis’ are of particular importance, since they aim to provide guidance on design, choice of outcomes, presentation of results and other methodological aspects. This way, it is hoped that research in the field of work outcomes in these diseases will be supported and harmonised, ensuring high quality and standards.
